# Assessing the association of leukocyte telomere length with ankylosing spondylitis and rheumatoid arthritis: A bidirectional Mendelian randomization study

**DOI:** 10.3389/fimmu.2023.1023991

**Published:** 2023-03-24

**Authors:** Donglei Wei, Yage Jiang, Jianwen Cheng, Hui Wang, Ke Sha, Jinmin Zhao

**Affiliations:** ^1^ Department of Orthopedic Trauma and Hand Surgery, The First Affiliated Hospital of Guangxi Medical University, Nanning, Guangxi, China; ^2^ Guangxi Key Laboratory of Regenerative Medicine, Orthopaedic Department, The First Affiliated Hospital of Guangxi Medical University, Nanning, Guangxi, China; ^3^ Department of Anesthesiology, The First Affiliated Hospital of Guangxi Medical University, Nanning, Guangxi, China

**Keywords:** leukocyte telomere length, ankylosing spondylitis, rheumatoid arthritis, Mendelian randomization, single nucleotide polymorphism

## Abstract

**Background:**

Telomere length shortening can cause senescence and apoptosis in various immune cells, resulting in immune destabilization and ageing of the organism. In this study, we aimed to systematically assess the causal relationship of leukocyte telomere length (LTL) with ankylosing spondylitis (AS) and rheumatoid arthritis (RA) using a Mendelian randomization study.

**Methods:**

LTL (n=472174) was obtained from the UK Biobank genome-wide association study pooled data. AS (n=229640), RA (n=212472) were obtained from FinnGen database. MR-Egger, inverse variance weighting, and weighted median methods were used to estimate the effects of causes. Cochran’s Q test, MR Egger intercept test, MR-PRESSO, leave-one-out analysis, and funnel plots were used to look at sensitivity, heterogeneity, and multiple effects. Forward MR analysis considered LTL as the exposure and AS, RA as the outcome. Reverse MR analysis considered AS, RA as the exposure and LTL as the outcome.

**Results:**

In the forward MR analysis, inverse variance-weighted and weighted median analysis results indicated that longer LTL might be associated with increased risk of AS (IVW: OR = 1.55, 95% CI: 1.14-2.11, p = 0.006). MR Egger regression analysis showed no pleiotropy between instrumental variables (IVs) (Egger intercept= 0.008, p = 0.294). The leave-one-out analysis showed that each single nucleotide polymorphism (SNP) of AS was robust to each outcome. No significant causal effects were found between AS, RA and LTL in the reverse MR analysis.

**Conclusion:**

Longer LTL may be related with an increased risk of developing AS, and these findings provide a foundation for future clinical research on the causal association between LTL and AS.

## 1 Introduction

Telomeres are small, highly conserved DNA repetitive sequences (TTAGGG) at the ends of eukaryotic cell linear chromosomes. These DNA components progressively shorten with each cell cycle and have an essential function in maintaining cellular chromosome stability. In most cell types, telomeres shorten with human age, eventually leading to replicative senescence (1, 2). Telomere length is usually referred to as leukocyte telomere length (LTL), which reflects both telomere length in other tissues and the senescence status of immune-related cells within the circulatory immune system (3). Although the relationship between leukocyte telomere length and disease is complex, LTL has been proposed as a marker of biological age and is associated with a high risk of multiple age-related diseases: including cardiovascular disease and cancer (4–6). In recent years, the involvement of telomere length, telomerase and protein complex systems in the pathogenesis of autoimmune diseases has become a hot research topic (7–9).

During the human immune response, immune cells grow exponentially and die when not needed, so they usually have extremely high replication rates. The telomeres within them are under tremendous stress. In addition to reflecting cell replication history, telomere shortening is influenced by factors such as oxidative stress and inflammation (10). As common autoimmune diseases, rheumatoid arthritis and ankylosing spondylitis, clinical and experimental data suggest that immune cells play an essential role in the pathogenesis of the diseases (11, 12). Furthermore, rheumatoid arthritis and ankylosing spondylitis may be related to single nucleotide polymorphisms (SNPs) (13). It was found that T lymphocytes from patients with rheumatoid arthritis and axial spondyloarthritis are susceptible to apoptosis due to mechanisms of abnormal telomere length or lack of upregulation of telomerase activity (11, 14, 15). However, as high-quality observational studies, we lack large samples of RCTs to explore whether a causal effect is associated with LTL and AS, RA.

Mendelian randomization (MR) is an instrumental variable (IV) analysis that uses SNPs as unconfounded proxies for exposure to estimate their effects on outcomes of interest. This reduces bias in observational epidemiological studies (16, 17). In MR analysis, according to Mendelian inheritance laws, SNPs are assumed to be randomly distributed in the general population, simulating the randomization process (18). Conceptually, MR is similar to RCT in that randomization occurs during meiosis and can be an essential strategy to strengthen causal inference when RCT is impractical or unethical (16). In the present study, we first performed a forward MR analysis to assess whether there was a causal effect between LTL as an exposure factor and AS, RA. Then, we performed a reverse MR analysis to assess whether there was an association with LTL using AS and RA as exposure factors. In this investigation, genetic variation as IV inferred a causal connection between outcome and exposure. Eliminating confounding factors and reverse causation effectively avoided bias in traditional epidemiological investigations (19). Furthermore, it may be more persuasive than traditional observational studies and bring fresh insights into treating and diagnosing AS and RA.

## 2 Materials and methods

### 2.1 Study design and data sources

Using summary data from genome-wide association studies (GWAS), we ran MR analysis to evaluate the bidirectional relationship of LTL with AS and RA. Data for the MR analyses were obtained from 2 GWAS public summary statistics databases containing mainly European ancestry. Pooled GWAS results for LTL were derived using genome-wide pooled data that screened 472,174 well-characterized adults in the UK Biobank (UKB), LTL quantified as telomere repeat copy ratios relative to single gene copy ratios, genetic variation in LTL GWAS adjusted for age and sex (4). The GWAS data for AS and RA were obtained from the FinnGen database, which collects and analyzes genomic and health data from 500,000 Finnish biobank participants. The AS, RA dataset contains 229,640 (2252 cases, 227,388 controls) and 212,472 (9855 cases, 202,617 controls) participants, respectively, and independent variant loci genetically associated with AS and RA were identified by comparison with the healthy population. Since all of the analyses in this paper were based on data that was available to the public, there was no need for an institutional review board to give ethical approval for this study.

### 2.2 Selection of instrumental variables for MR analysis

We performed stringent filtering steps to control SNP quality in two different GWAS pooled data. First, SNPs that were linked to the right exposure were chosen using genome-wide significance thresholds (p < 5 × 10-8). Second, SNPs that have a total linkage disequilibrium (LD, R2 ≥ 0.001 and 10 Mb). Third, to figure out the strength of genetic tools are, we left out SNPs with F-statistics less than 10. Lastly, SNPs that could be pleiotropic were taken out after MR-polynomial residuals and outliers (MR-PRESSO), and MR analysis was run again to see how stable it was. With the above screening criteria, we screened 8 and 14 SNPs as IVs when using the AS and RA dataset as exposure factors, respectively, and 92 SNPs as IVs when using the LTL dataset as instrumental variables. The SNPs utilized as instrumental variables are described in the supplementary file: **Tables S1–S3**.

### 2.3 Statistical analysis

In this study, we used the “TwoSampleMR” package in R (version 4.1.2) for the analysis. The odds ratio (OR) and 95% confidence interval (CI) were used to estimate the degree of causality for the binary outcomes. We employed three techniques of MR analysis (inverse variance weighting, weighted median, and MR Egger) to examine in both directions whether LTL is causally related to AS and RA. IVW was used to weigh the random variable measurements, using the inverse of the variance of each random variable, which minimizes the mean variance. Since random effects of IVW allow each SNP to produce different mean effects, we used inverse variance weighting as the primary method for MR analysis (20). However, as this method only yields accurate estimates when all genetic variants are valid instrumental variables, we complemented the regression method using MR Egger and weighted medians to assess the IVW method’s robustness (21, 22). The MR Egger regression intercept and 95% confidence interval (CI) were used to determine the degree of bias in the arbitrary estimates due to directional pleiotropy when 100% of the genetic variance was considered to be null IVs (21, 23). The weighted median, enables a consistent evaluation of causative effects when 50% of genetic variants are valid IVs (22).

There may be heterogeneity in intravenous fluids from different platforms or populations, which can influence the outcome. This study utilized Cochran’s Q test and funnelled plots to evaluate SNP heterogeneity. Horizontal pleiotropy is the association of genetic variations with various phenotypes in multiple pathways, which might render MR analysis ineffective (20). In order to assess unknown pleiotropy, we used several analytical approaches: First, sensitivity analyses used “leave one out” to explore the possibility that individual SNPs drive such causal associations. Second, MR Egger intercept tests were used to assess the pleiotropic association of genetic variants with other potential confounders. The regression intercept evaluates the magnitude of pleiotropy, and the closer the intercept is to 0, the less likely the gene is pleiotropic (21). This study used the bonferroni corrected P value (p< 0.025) as the significance threshold.

## 3 Results

### 3.1 Forward MR analysis: Causal effect of LTL on AS, RA

**Figure 1:**
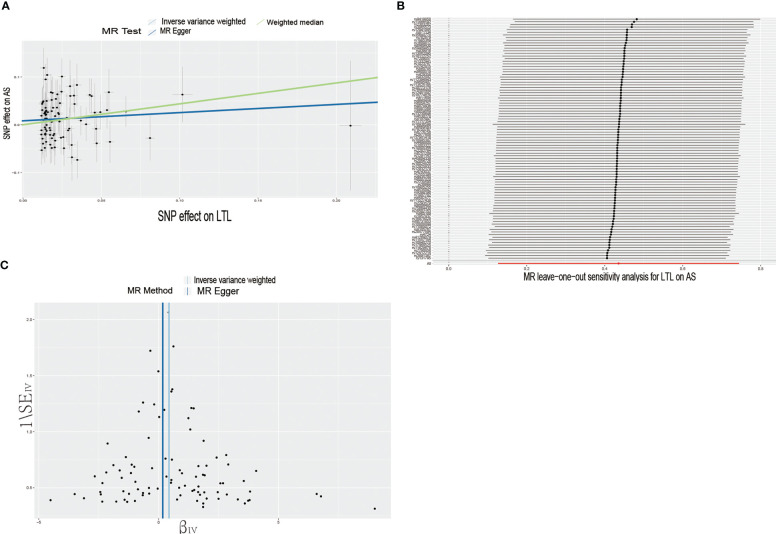
Causal effects of LTL on AS. **(A)** Scatter plot of the association between LTL and AS. This study used three methods to calculate the causal relationship between exposure factors and outcomes. The dark blue line represents MR Egger, the green line represents the weighted median, and the light blue line represents IVW. **(B)** Leave-one-out analysis to assess the effect of each SNP in driving causality. **(C)** Application of funnel plots to detect whether the observed associations are significantly heterogeneous. IVW, inverse variance weighting; LTL, leukocyte telomere length; AS, Ankylosing Spondylitis; MR, Mendelian randomization; SNP, single nucleotide polymorphism.

In the forward MR analysis, we analyzed the causal effect of LTL on AS and RA. The results of the MR analysis are shown in **Table 1**. The IVW results showed a significant association between LTL and ankylosing spondylitis (OR = 1.55, 95% CI: 1.14-2.11). The scatter plot of SNPs showing the effect of LTL on AS showed that the risk of AS was associated with a longer LTL (**Figure 1A**). Among the outcome variables in rheumatoid arthritis, the results of the IVW analysis method showed no significant association between LTL and RA (IVW: OR = 0.89, 95% CI: 0.75-1.05, p = 0.173, **Table 1**).

**Table 1:** MR Results of LTL Use on Risk of Ankylosing Spondylitis and Rheumatoid Arthritis.

Outcome	MR Methods	N SNPs	OR (95%CI)	Se	*P* value
AS	MR Egger	92	1.19(0.67-2.12)	0.30	0.562
	Weighted median	92	1.55(1.01-2.38)	0.22	0.045
	IVW	92	1.55(1.14-2.11)	0.16	0.006
RA	MR Egger	92	0.95(0.69-1.30)	0.13	0.746
	Weighted median	92	0.88(0.70-1.09)	0.10	0.242
	IVW	92	0.89(0.75-1.05)	0.07	0.173

AS, Ankylosing Spondylitis; RA, Rheumatoid Arthritis; LTL, Leukocyte Telomere Length; MR, mendelian randomization; IVW, inverse variance weighted; N SNPs, number of genetic instruments; OR, odds ratio; SNP, single nucleotide polymorphism.

To further investigate the association of LTL with AS and RA, we performed pleiotropy, heterogeneity and sensitivity analyses. The results showed no significant cross-sectional pleiotropy bias for the effects of LTL on AS and RA (AS: Egger intercept = 0.008, p = 0.294; RA: Egger intercept = -0.002, p = 0.644. **Table 2**). In addition, there was no significant heterogeneity between IVs for LTL effects on AS (Cochran Q = 117.15, p = 0.029, **Table 2**). Similarly, in the funnel plots of IVW and MR Egger, no significant heterogeneity between IVs was observed (**Figure 1C**). Significant heterogeneity was found between IVs for LTL effects on RA (Cochran Q = 142.51, p < 0.001, **Table 2**), however, as previously described, the random effects IVW approach allows for heterogeneity generated by SNPs. The leave-one-out method of sensitivity analysis showed that removing any of the 92 SNPs of AS did not significantly change the results (all rows were on the same side of 0) (**Figure 1B**), which shows that the MR analysis results were reliable.

**Table 2:** Heterogeneity and pleiotropy analysis in forward MR analysis.

Outcome	MR Methods	Cochran Q statistic	Egger intercept	Heterogeneity *p*-value	Pleiotropy *p*-value
AS	MR Egger	117.15	0.008	0.029	0.294
	IVW	118.60		0.028	
RA	MR Egger	142.51	-0.002	<0.001	0.644
	IVW	142.85		<0.001	

AS, Ankylosing Spondylitis; RA, Rheumatoid Arthritis; MR, mendelian randomization; IVW, inverse variance weighted.

### 3.2 Reverse MR analysis: Causal effect of AS, RA on LTL

In the reverse MR analysis, we analyzed whether AS and RA as exposure factors had a causal effect on LTL. The IVW results showed no significant association was found between AS, RA and LTL (**Table 3**). The results of the pleiotropic analysis showed a significant cross-sectional pleiotropic bias of RA on the IVs of LTL (Egger intercept = -0.0011, p = 0.006, **Table 4**). Therefore, the effect of RA on LTL may be influenced by other confounding factors, and there is a risk of false negatives.

**Table 3:** MR Results of Ankylosing Spondylitis and Rheumatoid Arthritis Used on the Effect of LTL.

Exposure	MR Methods	N SNPs	OR (95%CI)	Se	*P* value
AS	MR Egger	8	1.00(0.99-1.00)	0.004	0.828
	Weighted median	8	1.01(1.00-1.01)	0.003	0.110
	IVW	8	1.00(1.00-1.01)	0.002	0.069
RA	MR Egger	14	1.02(1.01-1.03)	0.005	0.001
	Weighted median	14	1.01(1.00-1.02)	0.004	0.011
	IVW	14	1.01(1.00-1.01)	0.004	0.060

AS, Ankylosing Spondylitis; RA, Rheumatoid Arthritis; LTL, Leukocyte Telomere Length; MR, mendelian randomization; IVW, inverse variance weighted; N SNPs, number of genetic instruments; OR, odds ratio; SNP, single nucleotide polymorphism.

**Table 4:** Heterogeneity and pleiotropy analysis in reverse MR analysis.

Exposure	MR Methods	Cochran Q statistic	Egger intercept	Heterogeneity *p*-value	Pleiotropy *p*-value
AS	MR Egger	3.04	0.002	0.804	0.312
	IVW	4.25		0.750	
RA	MR Egger	11.59	-0.001	0.479	0.006
	IVW	22.49		0.048	

AS, Ankylosing Spondylitis; RA, Rheumatoid Arthritis; MR, mendelian randomization; IVW, inverse variance weighted.

## 4 Discussion

With the improvement of medical treatment and the advancement of technology, the average life expectancy of human beings is increasing daily, and the aging problem is gradually attracting the medical community’s attention. A series of diseases such as aging-related malignancies, cardiovascular diseases, metabolic diseases and neurodegenerative pathologies have become essential research topics for scientists, and aging of the immune system plays a crucial role in them (4). Aging of the immune system can weaken the body’s ability to fight pathogenic microorganisms and kill tumor cells (24). On the other hand, increase the risk of developing autoimmune diseases, leading to a long-term chronic inflammatory state of the body (25). RA and AS, as chronic autoimmune diseases, both autoimmunity and auto-inflammation are involved in the pathogenesis of the disease (26–29). In this study, we used large-scale GWAS data from UK Biobank and Finnish Biobank participants to assess the possible causal relationship between LTL, AS, and RA by multiple MR methods. According to our research, in a European population, a longer LTL was related with an increased chance of developing AS. Reverse MR analysis revealed that genetically predicted AS and LTL have no causal link.

Ankylosing spondylitis, the most prevalent kind of spondyloarthritis, is a chronic inflammatory disease of the mid-axis spine that can manifest in a variety of clinical manifestations. Chronic back pain and growing rigidity of the spine are the most prevalent symptoms of the disease. AS is viewed as a combination of autoimmunity and autoinflammation. These components of innate immunity promote the onset of the disease, while the adaptive component is responsible for the continuation of the inflammatory process (29). In this investigation, we discovered that longer telomeres were related with an increased risk of AS.

Similarly, Tamayo et al. found in a cross-sectional research that patients with rheumatic illnesses had longer telomeres than controls (30). Four years later, the researchers continued their research on spondyloarthritis and discovered that individuals with rheumatic illnesses characterized by persistent systemic inflammation had longer peripheral blood leukocyte telomeres than controls (31). In contrast, this was not found in other rheumatic diseases without chronic systemic inflammation, such as osteoarthritis and osteoporosis (31). The mechanism behind this has not been fully clarified, and it has been suggested that chronic inflammation accompanying rheumatic diseases leads to this relative telomere lengthening effect. This may result from the breakdown of proteins of the sheltered complex or related factors associated with DNA repair or recombination (32). In addition, another study comprising 91 patients with Birdshot uveitis and 150 healthy controls revealed that the Birdshot patients had longer telomeres than the healthy controls, indicating a complicated telomere biology in chronic inflammation (33). Interestingly, like AS, Birdshot uveitis patients are closely associated with HLA class I (HLA-B27) (30, 31).

The ability of activated naive T cells to upregulate telomerase expression is also a possible explanation (34–37), but this ability remains contentious. In highly proliferative cells such as stem cells, germ cells, and the majority of cancer cells, telomerase plays a crucial role in telomere maintenance. In contrast to mature thymocytes, young nave T cells exhibit more telomerase production and activity during proliferation despite being normal somatic cells. They are barely detectable in mature resting naïve T cells (38, 39). Several studies have shown a slight increase in atopic abnormalities in AS patients compared to RA patients (40). Specific immunological pathways have been implicated in GWAS of this genetic disorder. These pathways include the IL-23/17 pathway, regulation of NF-kappaB activation, amino acid trimming of MHC antigen presentation, and genes that regulate CD8 and CD4 T cell populations (41). These findings imply that Th2 cells and chemokines may contribute to the development of AS (42). Interestingly, CD8+ antigen-specific T cells show a stronger inflammatory response in people with longer telomere lengths, and CD4+ antigen-specific T cells have longer telomere lengths than naive cells (43, 44). Lastly, the lengthening or shortening of leukocyte telomeres should not be considered a static event, but rather a dynamic one that occurs throughout time. The length of peripheral blood leukocyte telomeres may serve as a measure of chronic systemic inflammatory activity in AS, requiring subsequent pathological examination.

However, there are some concomitant limitations in our study. First, the data used in this study were primarily conducted on participants of European ancestry, the findings may be biased towards other ethnic groups with different lifestyles and cultural backgrounds. Secondly, we used data measuring TL GWAS in blood leukocytes, and LTL may not be sufficiently representative of telomere length in other cell or tissue subgroups associated with AS. Third, the uncertainty and incompatibility of sample sizes between the two major databases used in this study may simultaneously lead to some bias in our MR analysis. Fourth, MR evaluates inferred causal hypotheses by assigning genetic variants at random, it is challenging to discriminate between mediation and pleiotropy using MR methods alone. Numerous polymorphisms in the human genome may influence one or more phenotypes.

## 5 Conclusion

In conclusion, the present study found that longer LTL may be associated with an increased risk of AS. Our results suggest that LTL may be involved in the pathogenesis of AS. In the future, further studies are worthwhile to explore the correlation between LTL and AS in the pathogenesis and treatment strategies of AS.

## Data availability statement

Data from LTL are available in the UK Biobank (https://figshare.com/s/caa99dc0f76d62990195). Data for Ankylosing Spondylitis and Rheumatoid Arthritis were obtained from the FinnGen database (https://storage.googleapis.com/finngen-public-data-r7/summary_stats/finngen_R7_M13_ANKYLOSPON.gz and https://storage.googleapis.com/finngen-public-data-r7/summary_stats/finngen_R7_M13_RHEUMA.gz).

## Ethics statement

All of the analyses in this research are based on publicly accessible summary data, institutional review board approval was not necessary for this study.

## Author contributions

The final manuscript was read and approved by all writers. WD and JY designed the study, gathered and analyzed the data, and prepared and revised the final publication. WH and CJ collected and analyzed the data. SK and ZJ conceived the study and revised the text.
