# Supplementary material for: Assessing the association of leukocyte telomere length with ankylosing spondylitis and rheumatoid arthritis: A bidirectional Mendelian randomization study
Source: Front Immunol. 2023 Mar 24;14:1023991. doi: 10.3389/fimmu.2023.1023991 (PMC10080099; doi:10.3389/fimmu.2023.1023991)
Supplement: Supplementary file 1 [file Table_1.docx]

| **Supplementary Table 1: Genetic variants (n=92) of LTL used in MR analyses.** | | | | | | | |
| --- | --- | --- | --- | --- | --- | --- | --- |
| **SNPs** | **Effect allele** | **Other allele** | **Eaf** | **Beta** | **Se** | **pval** | **F** |
| rs429358 | T | C | 0.846 | -0.017 | 0.003 | 3.8E-10 | 10.214 |
| rs6751209 | T | C | 0.796 | 0.014 | 0.002 | 0.000000016 | 10.388 |
| rs6054257 | G | A | 0.206 | 0.014 | 0.002 | 0.000000011 | 10.719 |
| rs1003322 | C | A | 0.786 | -0.014 | 0.002 | 0.00000001 | 11.019 |
| rs41269079 | T | A | 0.811 | -0.015 | 0.003 | 1.7E-09 | 11.126 |
| rs11085072 | C | T | 0.763 | 0.013 | 0.002 | 0.000000026 | 11.210 |
| rs79755767 | G | A | 0.904 | -0.028 | 0.003 | 4.8E-16 | 11.406 |
| rs965109 | C | T | 0.976 | 0.102 | 0.006 | 1.8E-55 | 11.691 |
| rs2977608 | A | C | 0.256 | -0.013 | 0.002 | 0.00000003 | 11.694 |
| rs4660456 | A | G | 0.770 | -0.014 | 0.002 | 2.8E-09 | 12.489 |
| rs113525195 | C | A | 0.710 | 0.012 | 0.002 | 0.000000031 | 12.626 |
| rs4535042 | T | G | 0.701 | -0.012 | 0.002 | 0.000000033 | 12.806 |
| rs112394943 | T | C | 0.837 | 0.020 | 0.003 | 1.6E-12 | 13.600 |
| rs6587577 | A | G | 0.174 | 0.018 | 0.003 | 4.8E-12 | 13.705 |
| rs1907702 | G | A | 0.233 | -0.015 | 0.002 | 5.9E-10 | 13.713 |
| rs4743037 | C | T | 0.769 | -0.015 | 0.002 | 5.1E-10 | 13.717 |
| rs76666449 | T | C | 0.899 | -0.030 | 0.003 | 8.2E-19 | 14.201 |
| rs2282764 | A | G | 0.858 | 0.022 | 0.003 | 9.3E-15 | 14.663 |
| rs12451892 | T | C | 0.619 | 0.012 | 0.002 | 0.000000022 | 14.760 |
| rs6659669 | C | T | 0.395 | 0.012 | 0.002 | 0.000000011 | 15.566 |
| rs1957937 | A | T | 0.840 | -0.021 | 0.003 | 1.9E-14 | 15.782 |
| rs9600019 | C | T | 0.664 | -0.013 | 0.002 | 2.4E-09 | 15.873 |
| rs111527438 | T | C | 0.649 | -0.013 | 0.002 | 3.1E-09 | 15.993 |
| rs10805346 | T | C | 0.561 | -0.012 | 0.002 | 0.000000007 | 16.524 |
| rs7209057 | G | A | 0.439 | -0.012 | 0.002 | 5.7E-09 | 16.719 |
| rs38664 | T | C | 0.403 | 0.012 | 0.002 | 0.000000003 | 16.941 |
| rs10808899 | G | C | 0.111 | 0.030 | 0.003 | 3.1E-21 | 17.613 |
| rs13062095 | T | C | 0.672 | -0.014 | 0.002 | 9.7E-11 | 18.455 |
| rs2293579 | G | A | 0.614 | 0.013 | 0.002 | 3.3E-10 | 18.731 |
| rs10840270 | C | G | 0.344 | -0.014 | 0.002 | 1.3E-11 | 20.687 |
| rs10845387 | G | A | 0.647 | 0.014 | 0.002 | 1.5E-11 | 20.766 |
| rs13230646 | T | C | 0.751 | 0.017 | 0.002 | 8.9E-14 | 20.793 |
| rs12925933 | A | C | 0.338 | 0.015 | 0.002 | 7E-12 | 21.042 |
| rs869785 | T | C | 0.328 | 0.015 | 0.002 | 4.4E-12 | 21.108 |
| rs10773176 | A | G | 0.259 | 0.017 | 0.002 | 5.2E-14 | 21.734 |
| rs4530278 | G | T | 0.402 | -0.014 | 0.002 | 1.5E-11 | 21.893 |
| rs1023767 | G | A | 0.762 | 0.018 | 0.002 | 5E-15 | 22.189 |
| rs6007020 | T | C | 0.632 | -0.014 | 0.002 | 4.8E-12 | 22.220 |
| rs12932179 | A | G | 0.439 | 0.014 | 0.002 | 1.8E-11 | 22.240 |
| rs2555104 | A | C | 0.566 | 0.014 | 0.002 | 6.6E-12 | 23.163 |
| rs10024820 | T | C | 0.611 | 0.014 | 0.002 | 2.1E-12 | 23.473 |
| rs1985369 | A | G | 0.132 | 0.031 | 0.003 | 3.6E-25 | 24.584 |
| rs9878436 | C | T | 0.566 | 0.014 | 0.002 | 1.2E-12 | 24.812 |
| rs4695407 | A | G | 0.492 | -0.014 | 0.002 | 1.5E-12 | 25.045 |
| rs9398196 | A | G | 0.480 | 0.014 | 0.002 | 9.5E-13 | 25.431 |
| rs1332941 | A | G | 0.180 | -0.026 | 0.003 | 5.9E-21 | 25.988 |
| rs12941945 | A | G | 0.832 | 0.026 | 0.003 | 3E-22 | 26.252 |
| rs10774624 | G | A | 0.467 | -0.015 | 0.002 | 2.9E-13 | 26.508 |
| rs8102497 | G | A | 0.568 | 0.015 | 0.002 | 1.4E-13 | 26.847 |
| rs16978028 | A | T | 0.856 | 0.030 | 0.003 | 8.2E-26 | 27.161 |
| rs4498805 | G | T | 0.453 | -0.015 | 0.002 | 5.7E-14 | 28.000 |
| rs28577594 | G | C | 0.290 | -0.019 | 0.002 | 5.4E-17 | 28.907 |
| rs762679 | T | A | 0.143 | -0.031 | 0.003 | 1.4E-27 | 29.099 |
| rs66731853 | G | A | 0.683 | 0.018 | 0.002 | 1.5E-16 | 29.512 |
| rs2056726 | G | A | 0.786 | 0.023 | 0.002 | 7.9E-21 | 29.520 |
| rs11557154 | C | T | 0.870 | 0.034 | 0.003 | 1.1E-30 | 29.992 |
| rs59409453 | A | G | 0.269 | -0.020 | 0.002 | 1.6E-18 | 30.359 |
| rs6881568 | C | A | 0.637 | -0.017 | 0.002 | 3.7E-16 | 30.687 |
| rs2230590 | T | C | 0.489 | 0.016 | 0.002 | 3.6E-15 | 30.951 |
| rs131795 | A | T | 0.208 | 0.025 | 0.002 | 7.4E-24 | 33.383 |
| rs137901416 | G | A | 0.900 | -0.046 | 0.003 | 4.7E-43 | 34.159 |
| rs6776756 | G | A | 0.402 | 0.017 | 0.002 | 1.1E-17 | 35.257 |
| rs11866592 | G | A | 0.858 | -0.035 | 0.003 | 1.3E-33 | 35.592 |
| rs35640778 | G | A | 0.979 | 0.209 | 0.007 | 9.6E-195 | 36.030 |
| rs75664430 | C | G | 0.752 | 0.024 | 0.002 | 3.6E-24 | 38.380 |
| rs10905255 | G | T | 0.421 | 0.018 | 0.002 | 2.6E-19 | 39.359 |
| rs6669563 | G | A | 0.562 | -0.018 | 0.002 | 2.1E-19 | 39.932 |
| rs871134 | C | T | 0.431 | 0.018 | 0.002 | 1.7E-19 | 40.001 |
| rs45604339 | C | T | 0.658 | 0.020 | 0.002 | 4.3E-22 | 42.062 |
| rs5742915 | T | C | 0.554 | -0.019 | 0.002 | 1.6E-21 | 44.894 |
| rs8006485 | G | T | 0.547 | -0.019 | 0.002 | 1.2E-21 | 45.311 |
| rs11629678 | G | A | 0.671 | -0.022 | 0.002 | 1.2E-24 | 46.342 |
| rs17464525 | G | A | 0.819 | 0.032 | 0.003 | 6.6E-36 | 46.389 |
| rs11117354 | T | C | 0.303 | -0.023 | 0.002 | 3.4E-26 | 47.396 |
| rs6969930 | T | C | 0.387 | 0.020 | 0.002 | 1.4E-23 | 47.487 |
| rs12412214 | G | A | 0.720 | 0.025 | 0.002 | 3.4E-28 | 48.855 |
| rs28502153 | C | A | 0.622 | 0.022 | 0.002 | 1.2E-25 | 51.558 |
| rs932002 | C | T | 0.849 | 0.040 | 0.003 | 7.3E-47 | 52.950 |
| rs939916 | G | A | 0.330 | -0.024 | 0.002 | 6.6E-29 | 55.051 |
| rs4724 | G | A | 0.883 | 0.055 | 0.003 | 9.8E-69 | 63.253 |
| rs1609812 | G | A | 0.160 | -0.047 | 0.003 | 3.9E-65 | 78.173 |
| rs1291143 | A | C | 0.151 | -0.049 | 0.003 | 1.8E-69 | 79.586 |
| rs8105767 | A | G | 0.705 | -0.033 | 0.002 | 2.5E-50 | 92.533 |
| rs10112752 | G | A | 0.570 | 0.029 | 0.002 | 9.5E-46 | 98.848 |
| rs3891167 | A | G | 0.747 | 0.043 | 0.002 | 1.2E-70 | 119.523 |
| rs3865523 | G | T | 0.188 | 0.055 | 0.003 | 1.8E-93 | 128.404 |
| rs2303262 | C | T | 0.223 | 0.047 | 0.002 | 2.9E-85 | 132.690 |
| rs611646 | T | A | 0.591 | 0.037 | 0.002 | 3.5E-73 | 158.297 |
| rs7790856 | C | T | 0.711 | 0.044 | 0.002 | 1.8E-87 | 161.623 |
| rs9419958 | T | C | 0.139 | 0.081 | 0.003 | 2.6E-167 | 181.560 |
| rs4435700 | C | A | 0.235 | -0.054 | 0.002 | 2.2E-112 | 182.587 |
| rs10936598 | A | C | 0.676 | 0.066 | 0.002 | 1E-200 | 409.839 |
