# Supplementary material for: Assessing the association of leukocyte telomere length with ankylosing spondylitis and rheumatoid arthritis: A bidirectional Mendelian randomization study
Source: Front Immunol. 2023 Mar 24;14:1023991. doi: 10.3389/fimmu.2023.1023991 (PMC10080099; doi:10.3389/fimmu.2023.1023991)
Supplement: Supplementary file 2 [file Table_2.docx]

| **Supplementary Table 2: Genetic variants (n=8) of ankylosing spondylitis used in MR analyses.** | | | | | | | |
| --- | --- | --- | --- | --- | --- | --- | --- |
| **SNPs** | **Effect allele** | **Other allele** | **Eaf** | **Beta** | **Se** | **pval** | **F** |
| rs114799031 | T | A | 0.062 | 1.295 | 0.040 | 1.00E-200 | 119.938 |
| rs142695953 | A | C | 0.187 | 0.228 | 0.037 | 7.73E-10 | 11.489 |
| rs17190120 | T | G | 0.034 | 1.610 | 0.050 | 1.00E-200 | 68.721 |
| rs181316459 | C | G | 0.047 | 0.695 | 0.059 | 8.36E-32 | 12.292 |
| rs2032890 | C | A | 0.269 | -0.221 | 0.035 | 2.97E-10 | 15.595 |
| rs3130165 | G | C | 0.248 | 0.324 | 0.032 | 1.90E-23 | 37.158 |
| rs3804125 | A | G | 0.161 | 0.532 | 0.035 | 4.21E-51 | 61.179 |
| rs6759003 | C | T | 0.664 | -0.188 | 0.031 | 1.74E-09 | 16.183 |
