# Supplementary material for: Assessing the association of leukocyte telomere length with ankylosing spondylitis and rheumatoid arthritis: A bidirectional Mendelian randomization study
Source: Front Immunol. 2023 Mar 24;14:1023991. doi: 10.3389/fimmu.2023.1023991 (PMC10080099; doi:10.3389/fimmu.2023.1023991)
Supplement: Supplementary file 3 [file Table_3.docx]

| **Supplementary Table 3: Genetic variants (n=14) of rheumatoid arthritis used in MR analyses.** | | | | | | | |
| --- | --- | --- | --- | --- | --- | --- | --- |
| **SNPs** | **Effect allele** | **Other allele** | **Eaf** | **Beta** | **Se** | **pval** | **F** |
| rs35721478 | C | T | 0.128 | -0.171 | 0.023 | 1.92E-13 | 12.065 |
| rs4274624 | T | C | 0.769 | -0.105 | 0.017 | 1.29E-09 | 13.100 |
| rs8002731 | C | A | 0.363 | -0.089 | 0.016 | 1.24E-08 | 14.992 |
| rs7902904 | A | G | 0.530 | 0.084 | 0.015 | 2.43E-08 | 15.504 |
| rs706778 | T | C | 0.524 | 0.084 | 0.015 | 1.83E-08 | 15.798 |
| rs7731626 | A | G | 0.279 | -0.107 | 0.017 | 1.80E-10 | 16.350 |
| rs3087243 | A | G | 0.330 | -0.102 | 0.016 | 2.08E-10 | 17.866 |
| rs6456160 | C | T | 0.557 | -0.092 | 0.015 | 7.15E-10 | 18.747 |
| rs56240511 | C | G | 0.101 | 0.279 | 0.023 | 6.22E-34 | 26.871 |
| rs485502 | T | C | 0.606 | 0.166 | 0.015 | 1.54E-27 | 56.467 |
| rs2856822 | C | A | 0.254 | -0.253 | 0.018 | 5.49E-47 | 78.619 |
| rs2476601 | G | A | 0.851 | -0.347 | 0.019 | 2.00E-72 | 81.971 |
| rs56365848 | A | G | 0.103 | 0.487 | 0.022 | 6.89E-111 | 92.713 |
| rs9268839 | G | A | 0.440 | 0.454 | 0.014 | 1.00E-200 | 483.962 |
